# Molecular Identification of a Rare Subtype of *Cryptosporidium hominis* in Infants in China

**DOI:** 10.1371/journal.pone.0043682

**Published:** 2012-08-24

**Authors:** Huili Zhu, Jinfeng Zhao, Rongjun Wang, Longxian Zhang

**Affiliations:** 1 The College of Animal Science and Veterinary Medicine, Henan Agricultural University, Zhengzhou, China; 2 International Joint Research Laboratory for Zoonotic Diseases of Henan, Zhengzhou, China; Institut de Génétique et Microbiologie, France

## Abstract

Two *Cryptosporidium* isolates from separate infants suffering from diarrhea were obtained from a hospital in Zhengzhou, China and were genotyped by PCR amplification and sequence analysis of the small-subunit ribosomal RNA (rRNA) (SSU rRNA), 70-kDa heat shock protein (HSP70), and actin genes. Further subtyping was performed by PCR amplification and sequence analysis of the 60-kDa glycoprotein (gp60) gene. Both the isolates were identified as *Cryptosporidium hominis* subtype IdA21, a rare subtype previously found only in a human immunodeficiency virus-infected child in South Africa and another child in Jordan.

## Introduction

*Cryptosporidium*, a pathogenic protozoan parasite with a worldwide distribution, causes diarrhea in humans and animals. The parasite can be transmitted from person to person through fecal-oral contact (household contact and nosocomial transmission), ingestion of contaminated food or water, and contact with infected animals. In immunocompetent hosts, the infection is typically acute and self-limiting, whereas in immunocompromised individuals, such as persons taking immunosuppressive drugs and AIDS patients, the infection is often chronic [1].

Oocysts of *Cryptosporidium* species that are infectious to humans share similar morphology to those that are not, which prevents differentiation by light microscopy. Therefore, prevalence surveys based on oocyst morphology fail to determine the public health contribution of animals and environment to this human disease. Recent molecular epidemiologic studies of cryptosporidiosis have helped researchers gain a better understanding of human cryptosporidiosis transmission and the public health significance of *Cryptosporidium* spp. found in animals and the environment. With the use of genotyping tools, at least 23 valid species and numerous genotypes of *Cryptosporidium* have been described; eleven *Cryptosporidium* species (*C. hominis*, *C. parvum*, *C. meleagridis*, *C. felis*, *C. canis*, *C. ubiquitum*, *C. suis*, *C. muris*, *C. andersoni*, *C. cuniculus*, and *C. fayeri*) and at least five genotypes (skunk genotype, chipmunk genotype I, horse genotype, monkey genotype, and pig genotype II) can infect humans, with *C. hominis* and *C. parvum* being the most common clinical isolates [1]–[6]. By employing highly discriminatory subtyping techniques (generally sequence analysis of the gp60 gene), researchers have been able to track the infection source and the transmission dynamics of *C. hominis* and *C. parvum* [7].

Since the first report of human cryptosporidiosis in 1987 in Nanjing by Han et al., Xuzhou, Wuhu, and at least 17 other Chinese provinces or cities have reported this disease in their populations, with an infection rate ranging from 0.917% to 9.700% [8]–[10]. However, most of these studies were conducted using microscopy, and there is little available data on the molecular epidemiology of human cryptosporidiosis in China. *Cryptosporidium hominis* has been found to be the dominant infectious species, and three subtype families termed Ia, Ib, and Id have been identified. In contrast, only a few clinical isolates have been identified as *C. meleagridis*, *C. canis*, and *C. felis* [11]–[13]. To further characterize *Cryptosporidium* spp. in human clinical isolates in China, we performed a genotyping and subtyping study of two isolates of *C. hominis* by means of multilocus sequence typing.

**Figure 1:**
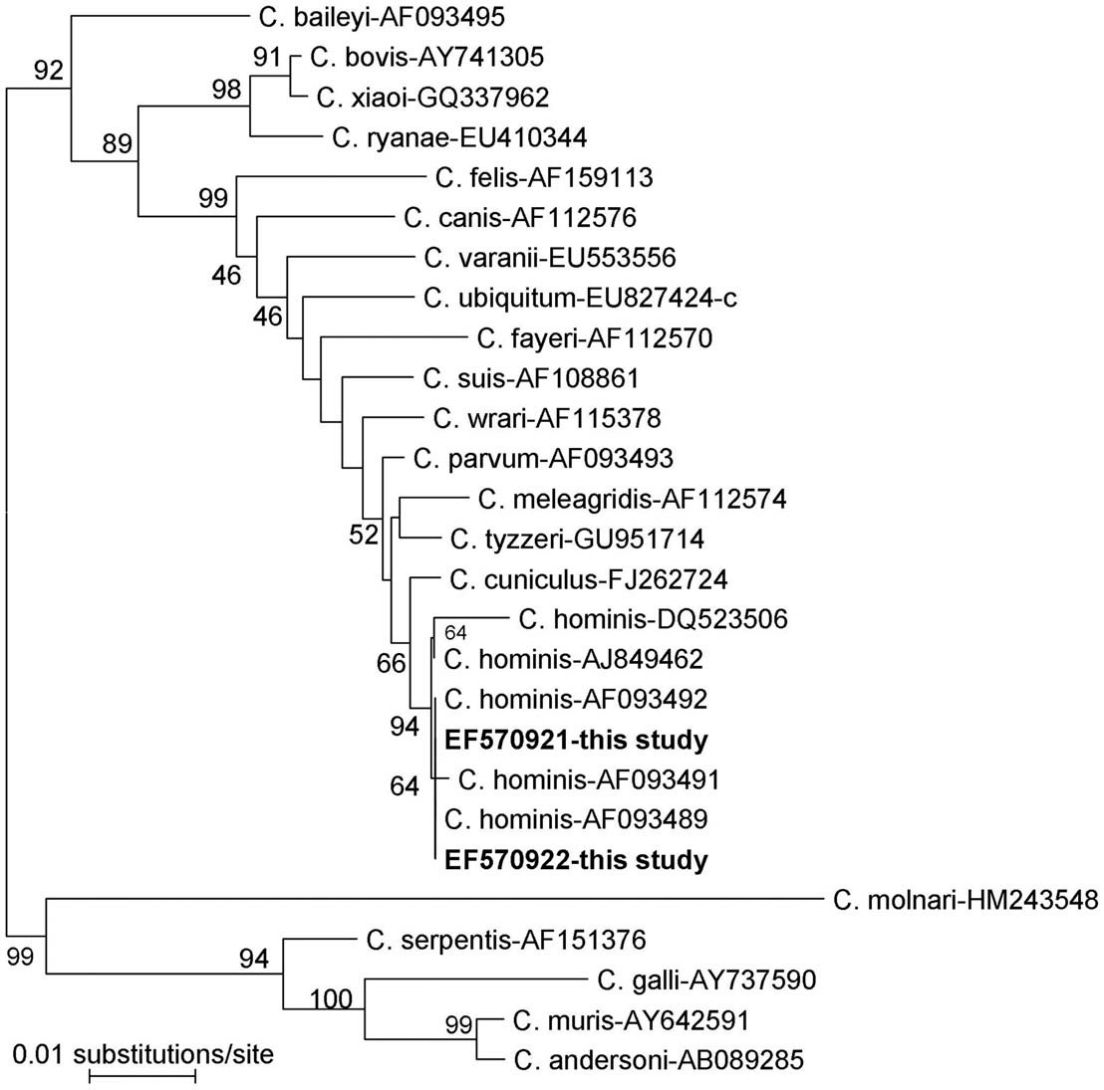
Phylogenetic relationship of *Cryptosporidium* parasites inferred by neighbor-joining analysis of the SSU rRNA based on evolutionary distances calculated using the Kimura two-parameter model. Bootstrap values were obtained using 1,000 pseudoreplicates.

## Materials and Methods

### *Cryptosporidium* Samples and DNA Extraction

Fecal samples were collected from clinical patients (including infants and those suffering from cancer) attending four hospitals in Zhengzhou, China. The samples were collected with the verbal consent of patients or their guardians. Since feces testing is a standard procedure in Chinese investigating hospitals, all patients agreed to sign the informed consent form. These completed forms were submitted to the ethics committee to examine and verify, in order to ensure the protection of the rights and benefits of patients. The study and this procedure were approved by the Research Ethics Committee of Henan Agricultural University. The stool samples of two infants less than one year old with a one week history of watery diarrhea were taken. The infants were diagnosed as *Cryptosporidium*-positive by bright field microscopy; the oocysts were concentrated by the Sheather’s sugar flotation technique and stored in a 2.5% potassium dichromate solution at 4°C until needed. Genomic DNA was isolated as previously described [14].

### Molecular Analyses

PCR amplification of the SSU rRNA, HSP70, actin and gp60 genes was performed as done in previous studies [1], [15]. RFLP analysis was performed on SSU rRNA PCR products; each sample was digested with *Ssp*I (Fermentas, USA) and *Vsp*I (Fermentas, USA), which produced a restriction pattern specific to *Cryptosporidium*. The PCR products of the SSU rRNA, HSP70 and gp60 genes, as well as the positive clones of actin gene, were sequenced by TaKaRa Biotechnology Co. Ltd (Dalian, China) on an ABI PRISMTM 3730 XL DNA Analyzer (Applied Biosystems, USA), using the Big Dye Terminator v3.1 Cycle Sequencing Kit (Applied Biosystems, USA).

Phylogenetic trees were constructed using the software PHYLIP (version 3.69). Neighbor-joining trees were constructed on the basis of evolutionary distances calculated using the Kimura two-parameter model. Sequence identity analysis was performed using the MegAlign program in the DNAStar software package.

The partial nucleotide sequences of the SSU rRNA, HSP70, actin and gp60 genes obtained in this study were deposited in the GenBank database under accession numbers EF570921 to EF570922 and EF591783 to EF591788.

## Results

DNA nucleotides of 837, 1951 and 1068 base pairs (bp) were obtained by PCR for the SSU rRNA, HSP70 and actin genes, respectively, from the two human isolates (CHZF1 and CHZF2). RFLP analysis of the SSU rRNA gene product with *Ssp*I and *Vsp*I showed restriction patterns identical to *C. hominis* [16]. The two *Cryptosporidium* isolates did not show any sequence differences in each gene. In the SSU rRNA gene, the sequence obtained was identical to AF093489 (from a USA isolate), AF108865 (from an Austria isolate), AJ849464 (from a Slovenia isolate), and DQ286403 (from a Chile isolate). However, it had 1–6 nucleotide differences with other *C. hominis* isolates (DQ523506, AF093492, AJ849462, EU331242 and AF093491), and the sequence identity varied from 99.3% to 99.9%. Similarly, in the HSP70 gene, three nucleotide changes (two C to T at nucleotide 4 and 1900, and a T to A at nucleotide 1903) and one nucleotide change (a C to T change at nucleotide 1912) were respectively observed for two different *C. hominis* isolates (AF401506 and AF401504). The nucleotide similarities with these two isolates were 99.8% and 99.9%, respectively. Interestingly, however, no nucleotide change was found in the actin gene when compared to other *C. hominis* isolates.

Neighbor-joining trees were constructed from the aligned partial SSU rRNA, HSP70 and actin sequences of these two *C. hominis* isolates, as well as those downloaded from the GenBank database. In the actin locus, the two isolates formed a cluster with *C. hominis*, and this was supported by bootstrap analysis. However, in the SSU rRNA and HSP70 genes, the two isolates clustered together and formed a sister clade with *C. hominis* (a neighbor-joining tree of the SSU rRNA gene is shown in Figure 1).

The 533 bp nucleotide sequence of the gp60 gene was amplified by nested PCR, and the two isolates yielded the same sequence and the subtype identity as established by sequence alignment and phylogenetic analysis: both isolates belonged to the subtype IdA21. In comparison with the previous reported Chinese *C. hominis* isolate IdA14 (AF403169), seven more TCA repeats was found, with the exception of a T to C change at nucleotide 11, and the isolates shared a nucleotide similarity of 95.6%.

## Discussion

SSU rRNA, HSP70, and actin genes are common molecular markers for genotyping and have been very valuable when used in conjunction with morphological, biological, or host specificity studies [1]. In this study, although there were no nucleotide changes found between the two isolates and other *C. hominis* isolates at the actin gene, sequence diversity was observed in the SSU rRNA and HSP70 genes when compared to other *C. hominis* isolates. Five copies of the SSU rRNA gene are present in the *Cryptosporidium* genome, and previous studies have suggested that there is slight sequence heterogeneity in some of these copies [17]. Therefore, some of the sequence differences might be attributed to the intragenomic variation of the multiple rRNA loci. However, the sequence differences found in the HSP70 gene between isolates could be due to a single nucleotide polymorphism (SNP).

In the present study, two *Cryptosporidium*-positive isolates were both found to belong to *C. hominis* based on sequence analyses of three genes. Previous studies suggest *C. parvum* and *C. hominis* are responsible for greater than 90% of human cases of cryptosporidiosis in most areas; however, the contribution of each species varies among different geographic regions [7]. In the United Kingdom, other parts of Europe, and New Zealand, *C. parvum* is responsible for slightly more infections than *C. hominis*. In contrast, *C. hominis* is responsible for far more infections than *C. parvum* in the United States, Australia, and Japan, as well as developing countries where genotyping studies have been conducted. Major differences in the transmission routes may be responsible for these observed differences in the distribution of *Cryptosporidium* species [7], [18]. In China, to the best of our knowledge, more than 100 *Cryptosporidium* isolates have been genotyped, and most of them were identified as *C. hominis* [11]–[13]. Thus, the predominant infectious *C. hominis* found in humans indicated human-to-human transmissions of *Cryptosporidium* spp. might play an important role in China. However, since exposure information was not gathered, it is difficult to determine the infection sources.

Ia, Ib and Id are the three common *C. hominis* subtype families, and each has been found in humans in many areas. Nevertheless, there are geographic differences in the distribution of subtype families. For example, Ib was a predominant *C. hominis* subtype family in humans in *C. parvum*-endemic areas such as Portugal, United Kingdom, and Australia [18], [19]. Both Ib and Id were common in developing countries such as Malawi, South Africa, India and Peru [18], [20], [21]. In New Orleans, Ib and Ia were both common, while Id subtypes were absent [18]. In the present study, the two *C. hominis* isolates were identified as IdA21, a relatively rare subtype that has only been detected in South Africa and Jordan [22], [23]. Id is one of the predominant subtype families found in China: Ia (35/95), Id (40/95) and Ib (7/95) have been identified in humans in Shanghai and Zhengzhou [12], [13]. However, due to the absence of genetic data of clinical *C. hominis* isolates in other areas, the population genetic structure of *C. hominis* subtypes is still unclear.

Further dissection of the infection sources and transmission dynamics of human cryptosporidiosis in China remains an important area of future investigation.
